# Hippocampal *LMNA* Gene Expression is Increased in Late-Stage Alzheimer’s Disease

**DOI:** 10.3390/ijms20040878

**Published:** 2019-02-18

**Authors:** Iván Méndez-López, Idoia Blanco-Luquin, Javier Sánchez-Ruiz de Gordoa, Amaya Urdánoz-Casado, Miren Roldán, Blanca Acha, Carmen Echavarri, Victoria Zelaya, Ivonne Jericó, Maite Mendioroz

**Affiliations:** 1 Neuroepigenetics Laboratory-Navarrabiomed, Complejo Hospitalario de Navarra, Universidad Pública de Navarra (UPNA), IdiSNA (Navarra Institute for Health Research), Pamplona, Navarra 31008, Spain; idoia.blanco.luquin@navarra.es (I.B.-L.); jsruizdegordoa@gmail.com (J.S.-R.d.G.); aurdanoz.1@gmail.com (A.U.-C.); miren.roldan.arrastia@navarra.es (M.R.); blancacha02@hotmail.com (B.A.); carmentxu.ez@gmail.com (C.E.); 2 Department of Internal Medicine, Hospital García Orcoyen, Estella 31200, Spain; 3 Department of Neurology, Complejo Hospitalario de Navarra- IdiSNA (Navarra Institute for Health Research), Pamplona, Navarra 31008, Spain; ivonne.jerico.pascual@navarra.es; 4 Hospital Psicogeriátrico Josefina Arregui, Alsasua, Navarra 31800, Spain; 5 Department of Pathology, Complejo Hospitalario de Navarra- IdiSNA (Navarra Institute for Health Research), Pamplona, Navarra 31008, Spain; mv.zelaya.huerta@navarra.es

**Keywords:** lamins, LMNA, Alzheimer’s disease, hippocampus, tauopathy, aging, lamin A, lamin C

## Abstract

Lamins are fibrillary proteins that are crucial in maintaining nuclear shape and function. Recently, B-type lamin dysfunction has been linked to tauopathies. However, the role of A-type lamin in neurodegeneration is still obscure. Here, we examined A-type and B-type lamin expression levels by RT-qPCR in Alzheimer’s disease (AD) patients and controls in the hippocampus, the core of tau pathology in the brain. *LMNA*, *LMNB1,* and *LMNB2* genes showed moderate mRNA levels in the human hippocampus with highest expression for the *LMNA* gene. Moreover, *LMNA* mRNA levels were increased at the late stage of AD (1.8-fold increase; *p*-value < 0.05). In addition, a moderate positive correlation was found between age and *LMNA* mRNA levels (Pearson’s *r* = 0.581, *p*-value = 0.018) within the control hippocampal samples that was not present in the hippocampal samples affected by AD. A-type and B-type lamin genes are expressed in the human hippocampus at the transcript level. *LMNA* mRNA levels are up-regulated in the hippocampal tissue in late stages of AD. The effect of age on increasing *LMNA* expression levels in control samples seems to be disrupted by the development of AD pathology.

## 1. Introduction

The nuclear lamina is a reticular structure attached to the internal nuclear membrane. It is essential for the maintenance of nuclear architecture and morphology and participates in critical cellular functions, such as DNA replication, transcription, chromatin organization, nuclear differentiation, or signal transduction, among others. 

In humans, the nuclear lamina is composed of three proteins. The *LMNA* gene encodes A-type lamin: lamin A/C, whereas *LMNB1* and *LMNB2* genes encode B-type lamins: lamin B1 and lamina B2 respectively. More than 20 human diseases have been related to mutations in these genes. *LMNA* gene mutations cause a wide spectrum of disorders involving muscle, adipose, bone, and peripheral nervous tissues along with premature aging syndromes, e.g., Werner syndrome (WS) and Hutchinson–Gilford progeria syndrome (HGPS) [1]. In addition, *LMNB1* gene duplication causes autosomal dominant leukodystrophy with autonomic disease (ADLD) [2] whereas homozygous missense mutations in the *LMNB2* gene have been associated with an acquired partial lipodystrophy called Barraquer–Simons syndrome and with progressive myoclonus epilepsy with early ataxia [3,4].

While mutations on B-type lamins cause cognitive decline, evidence linking A-type lamin to neurodegenerative diseases in humans is still quite scarce. On the one hand, it is recognized that dementia is not a feature usually found in *LMNA*-linked progeria disorders even though these syndromes mimic physiological aging at an early age [5]. In fact, the rate of brain aging is significantly lower than the rate of whole body aging in WS and HGPS [6]. Moreover, these patients do not show the typical neuropathological changes of Alzheimer’s disease (AD), such as amyloid plaques and neurofibrillary tangles [5,7]. On the other hand, a genome-wide association study (GWAS) revealed that a single nucleotide polymorphism in the *LMNA* gene (rs505058) increased the risk of late-onset AD [8], although this result is controversial as it was not confirmed in further studies [9,10,11]. 

More recently, Frost et al. described for the first time the relationship between B-type lamins and tauopathies, which are age-related neurodegenerative disorders characterized by abnormal protein tau deposits in the brain [12]. In a transgenic human tau *Drosophila* model, the authors observed that tau-induced lamin dysfunction leads to heterochromatin relaxation and neuronal cell death [13]. Aberrant phosphorylation of the tau protein would induce structural alterations of the nuclear envelope, including nuclear invaginations, identical to those observed in laminopathies by electron microscopy [13]. These morphological features were also observed in *postmortem* human brains of patients diagnosed with AD [13]. Interestingly, toxic accumulation of mRNA has also been observed within and adjacent to tau-induced nuclear envelope invaginations in a *Drosophila* model of tauopathy [14]. 

Here, we wanted to gain insight into the relationship between lamin genes expression and AD, as a paradigm of a neurodegenerative disease. To that end, we profiled mRNA expression levels of *LMNA*, *LMNB1,* and *LMNB2* genes by real time quantitative PCR (RT-qPCR) in the human hippocampus, which is one of the most vulnerable brain regions to AD and the core of pathological protein tau deposits [15]. To increase AD specificity, we used frozen *postmortem* hippocampal samples obtained from a cohort of pure AD cases showing only deposits of phosphorylated tau and β-amyloid along with controls free of any protein inclusion. 

## 2. Results

### 2.1. Lamin mRNA Levels across Alzheimer’s Disease Stages in the Human Hippocampus

First, we measured mRNA expression levels of *LMNA*, *LMNB1,* and *LMNB2* genes by RT-qPCR in human hippocampal samples from AD patients compared to controls. Two samples (AD patients) did not pass the RNA quality threshold so were not included in the experiments (see Methods section). Eventually, 28 AD cases were compared to 16 controls. Hippocampal mRNA expression levels (percentage of relative expression) differed significantly among the three genes (*F* = 38.905; *p*-value < 0.0001), as shown in Figure S1, with highest mRNA expression levels for the *LMNA* gene. Regarding disease status, no differences were found in the mRNA levels of *LMNA* (*p*-value = 0.824), *LMNB1* (*p*-value = 0.732), and *LMNB2* (*p*-value = 0.386) between AD cases and controls, as shown in Figure S2. 

**Figure 1:**
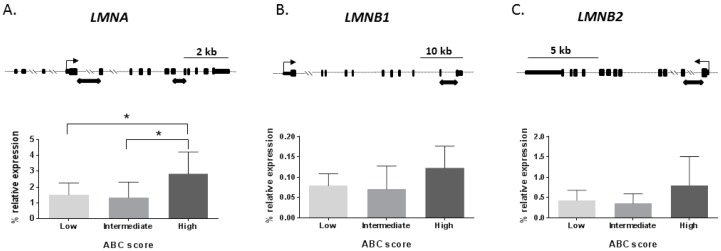
mRNA levels of lamin genes across Alzheimer’s disease (AD) stages. AD hippocampal samples were sorted in three levels [low (*n* = 14), intermediate (*n* = 6), and high (*n* = 8)] of AD neuropathological severity based on ABC score. According to that, mRNA expression levels are shown for each group and gene by bar graphs. The upper row shows the map for each gene. Where black squares represent exons, the thin arrow represents transcription start site for each gene; black rectangular arrow-boxes below the gene map denote amplicons of the RT-qPCR. (**A**) The *LMNA* gene graph shows a significant increase in expression among ABC stages. (**B**) A statistical tendency (*p*-value = 0.052) across ABC stages was found for the *LMNB1* gene, the high ABC stage being the one with highest expression. (**C**) The *LMNB2* gene did not show any difference between ABC stages. Boxes represent percentage of lamin genes expression relative to the geometric mean of *GAPDH* and *ACTB* housekeeping genes expression levels. Bars represent the standard error of the mean. * *p*-value < 0.05.

Next, a disease-staging analysis was performed to investigate changes of lamin genes’ mRNA levels depending on AD severity measured by ABC score staging [16]. ABC score combines histopathologic assessments of β-amyloid deposits determined by the method of Thal (A) [17], staging of neurofibrillary tangles by Braak and Braak classification (B) [18], and scoring of neuritic plaques by the method of CERAD (Consortium to Establish A Registry for Alzheimer’s Disease) (C) [19] to better characterize AD neuropathological changes [16]. Thus, the ABC score shows three levels of AD neuropathological severity: low, intermediate, and high. We found that *LMNA* mRNA levels significantly changed across ABC stages (*p*-value < 0.05) as shown in Figure 1A, with the maximum expression for the high ABC stage. The Scheffé post-hoc analysis revealed that *LMNA* mRNA expression was significantly different between low and high ABC stages (1.8-fold increase; *p*-value < 0.05) and between intermediate and high ABC stages (2.1-fold increase *p*-value < 0.05), as shown in Figure 1A. 

*LMNB1* mRNA levels showed a statistical trend to be different across AD severity groups (*p*-value = 0.052), as shown in Figure 1B, being the high ABC stage in the group with top expression. Finally, *LMNB2* mRNA levels did not show significant differences among the AD severity groups (*p*-value = 0.104), as shown in Figure 1C.

De novo mutations in *LMNA*-related progeria syndromes result in transcription and accumulation of progerin, a transcript characterized by a 150 bp deletion in exon 11 of the *LMNA* gene [1]. The progerin transcript is also found in low amounts in healthy [20] and diseased human tissues [21], and increases with age. To make sure that our RT-qPCR analysis was specific to full-length *LMNA* transcript and progerin was not being amplified, we used primers overlapping exons 9 to 12 of the *LMNA* transcript described elsewhere [22] to perform a RT-PCR. The progerin band was not observed, neither in the controls nor the AD hippocampal samples, as shown in Figure S3.

### 2.2. Western-Blot of Lamin A/C in the Human Hippocampus Affected by AD

**Figure 2:**
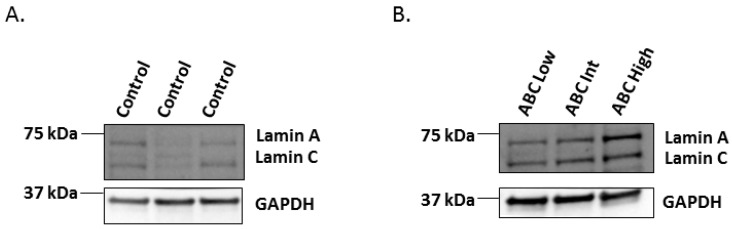
Western blot analysis of Lamin A/C proteins in human hippocampus. (**A**) Both lamin A and lamin C are expressed in control hippocampal samples. (**B**) Increasing tendency of lamin A and C protein expression from low to high ABC stages. GAPDH was used as a control. GAPDH: 37 kDa, Lamin A/C: 70-65 kDa.

To explore whether increased mRNA levels of *LMNA* in the high ABC stage of AD also affect protein levels, a Western blot analysis was completed. Protein extracts from frozen hippocampal samples included in the RT-qPCR experiment were obtained and a mouse monoclonal antibody which recognizes an epitope between residues 319–566 of pre-lamin A/C was used. This antibody reacts with both lamin A and lamin C proteins. Since lamin A protein expression is thought to be low in the brain and even lower compared to lamin C [23], we first tested whether both lamin A and lamin C were present in the normal human hippocampus. We found that control hippocampal samples showed similar protein expression levels for both lamin A and lamin C, as shown in Figure 2A. Next, we tested lamin A and lamin C protein expression levels across the three ABC stages of AD. In line with the results of *LMNA* mRNA levels, we observed that both lamin A and lamin C isoforms were increased in the high ABC stage of AD, as shown in Figure 2B. 

### 2.3. Correlation between Lamin Genes mRNA Levels and p-Tau Burden 

Next, we wanted to explore whether expression levels of the *LMNA* gene were related to hyperphosphorylated tau (p-tau) deposits in the hippocampus. To that end, we used a semi-automated quantitative method described in detail elsewhere [24] to measure the extension of p-tau deposits in our set of hippocampal samples. Since p-tau deposit values followed a bimodal distribution, Kendall’s tau-b test was calculated to evaluate the correlation between *LMNA* mRNA levels and the extension of p-tau deposits in the hippocampus. No significant correlation was found between p-tau burden and *LMNA* expression (tau-b = 0.153; *p*-value = 0.278).

### 2.4. Correlation between Lamin Genes mRNA Levels and Age 

**Figure 3:**
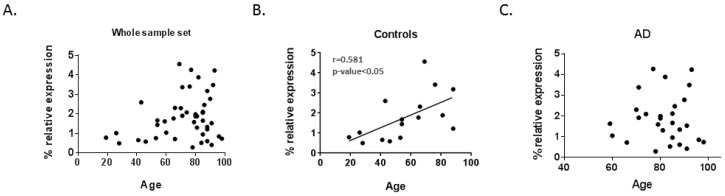
Scatter-plots showing the correlation level between *LMNA* mRNA levels and age. (**A**) No association was found between age and *LMNA* mRNA expression within the whole sample set (controls and Alzheimer’s patients). (**B**) Control group presented a medium significant positive correlation between *LMNA* mRNA levels and age. (**C**) No correlation within the AD group was observed between *LMNA* mRNA and age. Dots represent each sample according to percentage of *LMNA* expression relative to the geometric mean of *GAPDH* and *ACTB* housekeeping genes expression and their age. The straight line shows the regression line of *LMNA* mRNA levels and age. AD = Alzheimer’s disease.

In addition to *LMNA* gene mutations causing progeria syndromes characterized by premature aging, it has been described that *LMNA* mRNA expression increases with aging in human muscle [20] and is associated with specific age-related phenotypes in the normal population, such as vascular aging [25]. Moreover, whole genome sequencing and GWAS relate *LMNA* gene polymorphism rs915179 to extreme longevity [26,27]. Thus, we explored the relationship between lamin genes mRNA expression and age in our set of samples. When analyzing correlations between lamin gene expression levels and age for the global set of samples, no differences were found, as shown in Table 1 and Figure 3A. A statistically significant positive correlation was observed between *LMNA* mRNA levels and age within the group of controls, as shown in Table 1 and Figure 3B. However, this relationship was no longer shown in the AD group, as shown in Table 1, Figure 3C. 

## 3. Discussion 

An important finding of this study is that all lamin genes including *LMNA* show moderate mRNA expression levels in the human hippocampus. While no differences were found between controls and AD patients regarding any lamin gene expression, a statistically significant increase in *LMNA* mRNA levels was observed in the late stage of AD. Progerin transcript was not detected in our set of hippocampal samples. Finally, a positive correlation between age and *LMNA* mRNA levels was detected in the control group that was not present in the AD group. 

Although lamins are present in the nuclear lamina of every nucleated cell, mutations in their codifying genes do not cause functional or structural abnormalities across all human tissues [28]. For instance, *LMNA*-related progeria syndromes are characterized by premature aging of multiple tissues, such as cardiovascular, skin, bone, or adipose tissues [5,7]. Accumulation of a truncated form of lamin A, progerin, is toxic for these tissues [29,30]. However, the brain seems to be protected from this devastating aging acceleration [6,31]. Patients suffering from HGPS are spared from dementia and neuropathological changes of neurodegenerative diseases [5,7,31]. Nevertheless, this effect might be limited to the progeria-causing *LMNA* mutations since other *LMNA* mutations seem to induce cognitive decline or white matter lesions of the brain [32,33].

In any case, the scarce involvement of the central nervous system in progeria is still controversial [5] and different reasons have been argued to explain this effect. Among them, it has been reported that a brain-specific microRNA, miR-9, inhibits lamin A and progerin expression in neural cells generated from HGPS-derived induced pluripotent stem cells (iPSC) [34]. In a transgenic mouse model of HGPS, it was also shown that lamin A expression, but not lamin C, was down-regulated in the brain by miR-9 [35]. Therefore, it has been postulated that B-type lamins might be expressed at higher levels than A-type lamins in the brain [23]. However, expression of the *LMNA* gene in non-progeroid brain cells or tissue has not been well characterized. Our results suggest that *LMNA* expression at the mRNA level is not low in the human hippocampus. In fact, hippocampal *LMNA* mRNA levels showed lower Ct values in the RT-qPCR experiment than *LMNB1* and *LMNB2* mRNA levels. In addition, WB with a lamin A/C antibody revealed the presence of both lamin A and lamin C bands in the hippocampus of controls and AD patients. As for whether this expression pattern is extensible to the rest of human brain regions, that would be the subject of another study. 

A second finding of this study is that expression of the lamin genes is not different between AD patients and controls in the human hippocampus. This result would be in contrast to previous studies that showed a reduction in B-type lamins in tau transgenic human tau Drosophila model compared to wild type and in AD human brains compared to controls [13]. However, the latter study may have been underpowered since it was performed using ImageJ to measure immunofluorescence, which is a semi-quantitative approach and sample size was much lower than ours, as they compared six control brains versus six AD brains. In addition, the authors used human frontal cortex which is not the same brain region we used for our analysis. Having said that, we want to add that our result may be obscured by the characteristics of our control sample set since age was significantly different between AD and controls. Thus, this is a limitation of the present study which was focused on collecting pure AD cases and pure controls, the latter showing no abnormal protein inclusions. As a consequence of this stringent criterion, the number of old pure controls was limited in our sample set. 

Interestingly, we found an increase in *LMNA* mRNA levels in the human hippocampus at the late stage of AD. Despite AD being an age-related neurodegenerative disease, *LMNA*-related progeria syndrome patients are somewhat protected from AD neuropathological changes. Intriguingly, in a transgenic mouse model of HGPS, severe distortions of the nuclei of hippocampal neurons occurred, but no changes in gene expression, behavior, or neurogenesis were observed [36]. No evidence of inclusions or aberrant tau in brain sections was found either [36]. In any case, we could speculate that low levels of *LMNA* gene products in the brain, such as lamin A, may be protective against AD dementia. Thus, that idea would be in line with finding high levels of lamin A in the AD-affected brain. An alternative explanation for our result would be related with brain cell composition of the hippocampal samples. Since we are studying bulk hippocampal samples, we assume that the final results of lamin gene expression are representing the average of a given cell-type proportion. Thus, the results of our *LMNA* expression experiments may be influenced by cell-type relative proportion of the hippocampal samples across AD stages. In the late stages of AD, neuronal loss and astrogliosis are expected [37]. Thus, a lower proportion of neurons, which show miR-9-induced down-regulation of lamin A, may explain why global *LMNA* expression levels are higher in the high ABC score stage of AD. Future research dissecting the profile of different *LMNA* transcripts, including lamin A-specific and lamin C-specific transcripts, in the human hippocampus across AD stages may help to clarify some of these points.

Lamin A has also been implicated in physiological aging [38]. Notably, cell nuclei from old individuals show structural changes similar to those observed in *LMNA*-related progeria syndromes with lamin A disposition at the nuclear periphery and these changes are caused by sporadic use of the cryptic splice site that is constitutively activated in HGPS. Inhibition of this splice site reverse the age-related nuclear changes [38]. In our study, we found a positive correlation between age and *LMNA* mRNA levels within the set of control hippocampal samples. This outcome is consistent with previous reports of *LMNA* expression in skeletal muscle [20,39,40] that also found an age-related increase in expression of the *LMNA* transcript levels. Wegner et al. suggested that this may be a compensative response to neutralize age-related muscle deterioration [40]. That could also be the case in the aging brain. Interestingly, this correlation is lost in the hippocampal samples affected by AD, suggesting that AD pathogenic mechanisms might interfere with this physiological response in the aged brain. In particular, as high levels of *LMNA* transcript were only found at late stages of disease, it could be hypothesized that this change would be a consequence of the pathological processes occurring in AD and that the proposed compensative physiological response of *LMNA* would be lost as the disease progresses. Further research of the mechanistic nature will be needed to test this relationship. 

## 4. Materials and Methods

### 4.1. Human Hippocampal Samples and Neuropathological Examination

Brain hippocampal samples from 46 subjects (30 AD patients and 16 controls) were provided by Navarrabiomed Biobank. After death, half brain specimens from donors were cryopreserved at −80 °C. Neuropathological examination was completed following the usual recommendations [41] and according to the updated National Institute on Aging-Alzheimer’s Association guidelines by evaluating the ABC score staging [42]. Assessment of β-amyloid deposition was carried out by immunohistochemical staining of paraffin-embedded sections (3–5 µm thick) with a mouse monoclonal (S6F/3D) anti β-amyloid antibody (Leica Biosystems Newcastle Ltd., Newcastle upon Tyne, UK). Evaluation of neurofibrillary pathology was performed with a mouse monoclonal antibody anti-human PHF-TAU, clone AT-8 (Tau AT8), (Innogenetics, Ghent, Belgium), which identifies hyperphosphorylated tau (p-tau) [18]. The reaction product was visualized using an automated slide immunostainer Leica Bond Max (Leica Biosystems Newcastle Ltd, Newcastle upon Tyne, UK), with Bond Polymer Refine Detection (Leica Biosystems Newcastle Ltd, Newcastle upon Tyne, UK).

To avoid spurious findings related to multiprotein deposits, pure AD cases with deposits of only p-tau and β-amyloid were eligible for the study and controls were free of any pathological protein aggregate. This approach maximizes chances of finding true associations with AD, even though reducing the final sample size and the number of older controls. A summary of the characteristics of subjects included in the study is shown in Table 2. The group of control subjects showed a lower mean age compared to the other ABC scale groups (*p* < 0.01). No differences in age were found across the ABC scale groups (low to high). Gender and post-mortem interval (PMI) were similar among all study groups. 

### 4.2. Lamin mRNA Expression Analysis by RT-qPCR

Total RNA was isolated from hippocampal homogenates using RNeasy Lipid Tissue Mini kit (QIAGEN, Redwood City, CA, USA), following manufacturer’s instructions. Genomic DNA was removed with recombinant DNase (TURBO DNA-free™ Kit, Ambion, Inc., Austin, TX, USA). RNA integrity was checked by 1.25% agarose gel electrophoresis under denaturing conditions. Concentration and purity of RNA were both evaluated with a NanoDrop spectrophotometer. Only RNA samples showing a minimum quality index (260 nm/280 nm absorbance ratios between 1.8 and 2.2 and 260 nm/230 nm absorbance ratios higher than 1.8) were included in the study. Complementary DNA (cDNA) was reverse transcribed from 1500 ng total RNA with SuperScript^®^ III First-Strand Synthesis Reverse Transcriptase (Invitrogen, Carlsbad, CA, USA) after priming with oligo-d (T) and random primers. RT-qPCR reactions were performed in triplicate with Power SYBR Green PCR Master Mix (Invitrogen, Carlsbad, CA, USA) in a QuantStudio 12K Flex Real-Time PCR System (Applied Biosystems, Foster City, CA, USA) and repeated twice within independent cDNA sets. In addition, two different sets of primers were designed for the *LMNA* gene to increase the reliability of the result. Sequences of primer pairs were designed using the Real Time PCR tool (IDT, Coralville, IA, USA) as follows: LMNA_1 F5′-TGGAGGAGGTGGATGAGG-3′, R5′-CGGTAAGTCAGCAAGGGATC-3′; LMNA_2 F5′-AGACCCTTGACTCAGTAGCC-3′, R5′-AGCCTCCAGGTCCTTCA-3′; LMNB1 F5′-TGGAGTGGTTGTTGAGGAAG-3′, R5′- GAGAAGGCTCTGCACTGTATAC-3′; LMNB2 F5′-TGCGTGAGAATGAGAATGGG-3′; R5′-AAGAAAGGTGTGTGGATGAGG-3′. Relative expression mRNA levels of lamin genes in a particular sample were calculated as previously described [43] and the geometric mean of *ACTB* and *GAPDH* genes was used as the reference to normalize expression values.

### 4.3. Progerin mRNA Expression Analysis by RT-PCR

To explore whether *LMNA* 150 bp-deletion transcript, also referred to as progerin, was present in the human hippocampus, a reverse transcription-polymerase chain reaction (RT-PCR) was performed. Primers spanning exons 9 to 12 of the *LMNA* transcript which include the cryptic splice donor site that originates progerin in the amplicon as follows: Progerin_F5′- GTGGAAGGCACAGAACACCT-3′ and Progerin_R5′-GTGAGGAGGACGCAGGAA-3′, and RT-PCR conditions were used as describe elsewhere [22].

### 4.4. LMNA Protein Expression Analysis by Western Blot

To prepare total protein extract, human hippocampus tissue from patients and control samples was lysed with 100 μL lysis buffer containing urea, thiourea, and DTT (dithiothreitol). After centrifugation at 35,000 rpm for 1 h at 15 °C, protein quantification was determined following the Bradford-Protein Assay (BioRad, Hercules, CA, USA) using a spectrophotometer.

Next, 20 μg of protein per sample was electrophoresed on 4–15% Criterion TGX stain-free gels (Bio-Rad, Hercules, CA, USA) under reducing conditions and transferred onto nitrocellulose membranes using a Trans-blot Turbo transfer system (25 V, 7 min) (Bio-Rad, Hercules, CA, USA). Equal loading of the gel was assessed by stain free digitalization and Ponceau staining. Membranes were probed with mouse anti-human Lamin A + C primary antibody (Abcam, Cambridge, UK) (ab8984; 1:300) in 5% nonfat milk and incubated with peroxidase-conjugated anti-rabbit secondary antibody (Cell Signaling, Danvers, MA, USA)(1:2000). Immunoblots were then visualized by exposure to an enhanced chemiluminescence ECL Select™ Western Blotting Detection Reagent (Amersham, GE Healthcare, Chicago, IL, USA) using a ChemidocMP Imaging System (Bio-Rad). GAPDH (Calbiochem, San Diego, CA, USA) (1:10,000) was used as the control in each lane. 

### 4.5. Quantitative Assessment of P-Tau Deposits in Hippocampal Samples

To quantitatively assess p-tau burden in the hippocampal samples we applied a method to quantify protein deposits, as described in detail elsewhere [24]. In brief, hippocampal sections were examined after performing immunostaining with anti p-tau antibodies and p-tau deposits were analyzed with ImageJ software in order to obtain an average quantitative measure of the global p-tau deposit for each section.

### 4.6. Statistical Data Analysis

Statistical analysis was performed with SPSS 21.0 (IBM, Inc., Chicago, IL, USA). First, we checked that all continuous variables showed a normal distribution, as per the one-sample Kolmogorov–Smirnov test and the normal quantile-quantile (QQ) plots. Data represents the mean ± standard deviation (SD). Significance level was set at *p*-value < 0.05. Statistical significance for lamin gene mRNA levels between the AD and control groups was assessed by T-test. One-way analysis of variance (ANOVA) followed by the Scheffé post-hoc analysis was used to analyze differences in the expression levels of lamin gene mRNA across ABC stages. Levene’s test was conducted to assess homogeneity of variance. Kendall’s tau-b correlation coefficient was used to determine correlation between p-tau burden and *LMNA* mRNA expression levels. The Pearson product-moment correlation coefficient analysis was performed to correlate lamin gene mRNA expression levels with age. GraphPad Prism version 7.00 for Windows (GraphPad Software, La Jolla, CA, USA) was used to draw graphs.

### 4.7. Ethics Approval and Consent to Participate

The Ethics Committee of the “Complejo Hospitalario de Navarra” approved the use of human subjects for this study (Pyto 90/2014). Written informed consent was obtained from all subjects or next of kin.

## Figures and Tables

**Table 1:** Correlation between age and lamin gene expression.

	Total Sample Set (*n* = 44)		Controls (*n* = 16)		AD Patients (*n* = 28)	
Gene	*r* Coefficient	*p*-Value	*r* Coefficient	*p*-Value	*r* Coefficient	*p*-Value
*LMNA*	0.254	0.092	0.581*	0.018	0.002	0.990
*LMNB1*	0.211	0.164	0.149	0.582	0.358	0.061
*LMNB2*	0.222	0.143	0.396	0.129	0.165	0.401

AD = Alzheimer’s disease, *r* coefficient = Pearson’s coefficient, * *p*-value < 0.05.

**Table 2:** Summary of characteristics of subjects included in the analysis sorted by ABC score.

ABC Score Staging	Controls (*n* = 16)	ABC Low (*n* = 14)	ABC Intermediate (*n* = 6)	ABC High (*n* = 8)
Age at death (years)	56.1 ± 21.6	78.6 ± 9.4	88.3 ± 6.6	81.2 ± 12
Gender (Female) %	43.8	64.3	66.7	62.5
PMI (h)	7.5 ± 4.6	8.3 ± 8.6	4.9 ± 2.9	6.3 ± 4.4

PMI: post-mortem interval; h: hours.

## Supplementary Materials

The following are available online at http://www.mdpi.com/1422-0067/20/4/878/s1, Figure S1: Box-plot of percentage of relative expression values of *LMNA*, *LMNB1* and *LMNB2* genes, Figure S2: mRNA levels of lamin genes in AD and control groups, Figure S3: Agarose gel electrophoresis of Reverse Transcription-PCR products by using primers spanning LMNA exon 9-1.

## References

[1] Mendez-Lopez I., Worman H.J. (2012). Inner nuclear membrane proteins: Impact on human disease. Chromosoma.

[2] Padiath Q.S., Saigoh K., Schiffmann R., Asahara H., Yamada T., Koeppen A., Hogan K., Ptáček L.J., Fu Y.H. (2006). Lamin B1 duplications cause autosomal dominant leukodystrophy. Nat. Genet..

[3] Damiano J.A., Afawi Z., Bahlo M., Mauermann M., Misk A., Arsov T., Oliver K.L., Dahl H.H., Shearer A.E., Smith R.J. (2015). Mutation of the nuclear lamin gene LMNB2 in progressive myoclonus epilepsy with early ataxia. Hum. Mol. Genet..

[4] Hegele R.A., Cao H., Liu D.M., Costain G.A., Charlton-Menys V., Wilson Rodger N., Durrington P.N. (2006). Sequencing of the reannotated LMNB2 gene reveals novel mutations in patients with acquired partial lipodystrophy. Am. J. Hum. Genet..

[5] Coppede F. (2013). The epidemiology of premature aging and associated comorbidities. Clin. Interv. Aging.

[6] Isaev N.K., Genrikhs E.E., Oborina M.V., Stelmashook E.V. (2018). Accelerated aging and aging process in the brain. Rev. Neurosci..

[7] Ullrich N.J., Gordon L.B. (2015). Hutchinson-Gilford progeria syndrome. Handb. Clin. Neurol..

[8] Grupe A., Abraham R., Li Y., Rowland C., Hollingworth P., Morgan A., Jehu L., Segurado R., Stone D., Schadt E. (2007). Evidence for novel susceptibility genes for late-onset Alzheimer’s disease from a genome-wide association study of putative functional variants. Hum. Mol. Genet..

[9] Belbin O., Carrasquillo M.M., Crump M., Culley O.J., Hunter T.A., Ma L., Bisceglio G., Zou F., Allen M., Dickson D.W. (2011). Investigation of 15 of the top candidate genes for late-onset Alzheimer’s disease. Hum. Genet..

[10] Yeh H.L., Hou S.J., Yen F.C., Hong C.J., Liou Y.J., Yang A.C., Liu M.E., Tsai S.J. (2011). Polymorphisms in LMNA and near a SERPINA13 gene are not associated with cognitive performance in Chinese elderly males without dementia. Neurosci. Lett..

[11] Schjeide B.M., McQueen M.B., Mullin K., DiVito J., Hogan M.F., Parkinson M., Hooli B., Lange C., Blacker D., Tanzi R.E. (2009). Assessment of Alzheimer’s disease case-control associations using family-based methods. Neurogenetics.

[12] Black D.S., Cole S.W., Irwin M.R., Breen E., St Cyr N.M., Nazarian N., Khalsa D.S., Lavretsky H. (2013). Yogic meditation reverses NF-κB and IRF-related transcriptome dynamics in leukocytes of family dementia caregivers in a randomized controlled trial. Psychoneuroendocrinology.

[13] Frost B., Bardai F.H., Feany M.B. (2016). Lamin Dysfunction Mediates Neurodegeneration in Tauopathies. Curr. Biol..

[14] Cornelison G.L., Levy S.A., Jenson T., Frost B. (2018). Tau-induced nuclear envelope invagination causes a toxic accumulation of mRNA in Drosophila. Aging Cell.

[15] Lace G., Savva G.M., Forster G., de Silva R., Brayne C., Matthews F.E., Barclay J.J., Dakin L., Ince P.G., Wharton S.B. (2009). MRC-CFAS, Hippocampal tau pathology is related to neuroanatomical connections: An ageing population-based study. Brain.

[16] Braak H., Braak E. (1991). Neuropathological stageing of Alzheimer-related changes. Acta Neuropathol..

[17] Thal D.R., Rüb U., Orantes M., Braak H. (2002). Phases of A beta-deposition in the human brain and its relevance for the development of AD. Neurology.

[18] Braak H., Alafuzoff I., Arzberger T., Kretzschmar H., Del Tredici K. (2006). Staging of Alzheimer disease-associated neurofibrillary pathology using paraffin sections and immunocytochemistry. Acta Neuropathol..

[19] Mirra S.S., Heyman A., McKeel D., Sumi S.M., Crain B.J., Brownlee L.M., Vogel F.S., Hughes J.P., van Belle G., Berg L. (1991). The Consortium to Establish a Registry for Alzheimer’s Disease (CERAD). Part II. Standardization of the neuropathologic assessment of Alzheimer’s disease. Neurology.

[20] Luo Y.B., Mitrpant C., Johnsen R.D., Fabian V.A., Fletcher S., Mastaglia F.L., Wilton S.D. (2013). Investigation of age-related changes in LMNA splicing and expression of progerin in human skeletal muscles. Int. J. Clin. Exp. Pathol..

[21] Messner M., Ghadge S.K., Goetsch V., Wimmer A., Dorler J., Polzl G., Zaruba M.M. (2018). Upregulation of the aging related LMNA splice variant progerin in dilated cardiomyopathy. PloS ONE.

[22] Harhouri K., Navarro C., Baquerre C., Da Silva N., Bartoli C., Casey F., Mawuse G.K., Doubaj Y., Levy N., De Sandre-Giovannoli A. (2016). Antisense-Based Progerin Downregulation in HGPS-Like Patients’ Cells. Cells.

[23] Frost B. (2016). Alzheimer’s disease: An acquired neurodegenerative laminopathy. Nucleus.

[24] Celarain N., Sánchez-Ruiz de Gordoa J., Zelaya M.V., Roldán M., Larumbe R., Pulido L., Echavarri C., Mendioroz M. (2016). TREM2 upregulation correlates with 5-hydroxymethycytosine enrichment in Alzheimer’s disease hippocampus. Clin. Epigenet..

[25] Ragnauth C.D., Warren D.T., Liu Y., McNair R., Tajsic T., Figg N., Shroff R., Skepper J., Shanahan C.M. (2010). Prelamin A acts to accelerate smooth muscle cell senescence and is a novel biomarker of human vascular aging. Circulation.

[26] Sebastiani P., Riva A., Montano M., Pham P., Torkamani A., Scherba E., Benson G., Milton J.N., Baldwin C.T., Andersen S. (2011). Whole genome sequences of a male and female supercentenarian, ages greater than 114 years. Front. Genet..

[27] Sebastiani P., Solovieff N., Dewan A.T., Walsh K.M., Puca A., Hartley S.W., Melista E., Andersen S., Dworkis D.A., Wilk J.B. (2012). Genetic signatures of exceptional longevity in humans. PloS ONE.

[28] Worman H.J., Schirmer E.C. (2015). Nuclear membrane diversity: Underlying tissue-specific pathologies in disease?. Curr. Opin. Cell Biol..

[29] De Sandre-Giovannoli A., Bernard R., Cau P., Navarro C., Amiel J., Boccaccio I., Lyonnet S., Stewart C.L., Munnich A., Le Merrer M. (2003). Lamin a truncation in Hutchinson-Gilford progeria. Science.

[30] Eriksson M., Brown W.T., Gordon L.B., Glynn M.W., Singer J., Scott L., Erdos M.R., Robbins C.M., Moses T.Y., Berglund P. (2003). Recurrent de novo point mutations in lamin A cause Hutchinson-Gilford progeria syndrome. Nature.

[31] Young S.G., Jung H.J., Coffinier C., Fong L.G. (2012). Understanding the roles of nuclear A- and B-type lamins in brain development. J. Biol. Chem..

[32] Bonati U., Bechtel N., Heinimann K., Rutz E., Schneider J., Frank S., Weber P., Fischer D. (2014). Congenital muscular dystrophy with dropped head phenotype and cognitive impairment due to a novel mutation in the LMNA gene. Neuromuscul. Disord..

[33] Hattori A., Komaki H., Kawatani M., Sakuma H., Saito Y., Nakagawa E., Sugai K., Sasaki M., Hayashi Y.K., Nonaka I. (2012). A novel mutation in the LMNA gene causes congenital muscular dystrophy with dropped head and brain involvement. Neuromuscul. Disord..

[34] Nissan X., Blondel S., Navarro C., Maury Y., Denis C., Girard M., Martinat C., De Sandre-Giovannoli A., Levy N., Peschanski M. (2012). Unique preservation of neural cells in Hutchinson- Gilford progeria syndrome is due to the expression of the neural-specific miR-9 microRNA. Cell Rep..

[35] Jung H.J., Coffinier C., Choe Y., Beigneux A.P., Davies B.S., Yang S.H., Barnes R.H., Hong J., Sun T., Pleasure S.J. (2012). Regulation of prelamin A but not lamin C by miR-9, a brain-specific microRNA. Proc. Natl. Acad. Sci. USA.

[36] Baek J.H., Schmidt E., Viceconte N., Strandgren C., Pernold K., Richard T.J., Van Leeuwen F.W., Dantuma N.P., Damberg P., Hultenby K. (2015). Expression of progerin in aging mouse brains reveals structural nuclear abnormalities without detectible significant alterations in gene expression, hippocampal stem cells or behavior. Hum. Mol. Genet..

[37] De Strooper B., Karran E. (2016). The Cellular Phase of Alzheimer’s Disease. Cell.

[38] Scaffidi P., Misteli T. (2006). Lamin A-dependent nuclear defects in human aging. Science.

[39] Pedersen K.M., Finsen B., Celis J.E., Jensen N.A. (1998). Expression of a novel murine phospholipase D homolog coincides with late neuronal development in the forebrain. J. Biol. Chem..

[40] Wegner L., Anthonsen S., Bork-Jensen J., Dalgaard L., Hansen T., Pedersen O., Poulsen P., Vaag A. (2010). LMNA rs4641 and the muscle lamin A and C isoforms in twins—Metabolic implications and transcriptional regulation. J. Clin. Endocrinol. Metab..

[41] Bell J.E., Alafuzoff I., Al-Sarraj S., Arzberger T., Bogdanovic N., Budka H., Dexter D.T., Falkai P., Ferrer I., Gelpi E. (2008). Management of a twenty-first century brain bank: Experience in the BrainNet Europe consortium. Acta Neuropathol..

[42] Montine T.J., Phelps C.H., Beach T.G., Bigio E.H., Cairns N.J., Dickson D.W., Duyckaerts C., Frosch M.P., Masliah E., Mirra S.S. (2012). National Institute on Aging-Alzheimer’s Association guidelines for the neuropathologic assessment of Alzheimer’s disease: A practical approach. Acta Neuropathol..

[43] Livak K.J., Schmittgen T.D. (2001). Analysis of relative gene expression data using real-time quantitative PCR and the 2(-Delta Delta C(T)) Method. Methods.

