# Supplementary material for: Hippocampal LMNA Gene Expression is Increased in Late-Stage Alzheimer’s Disease
Source: Int J Mol Sci. 2019 Feb 18;20(4):878. doi: 10.3390/ijms20040878 (PMC6413092; doi:10.3390/ijms20040878)
Supplement: Supplementary file 1 [file ijms-20-00878-s001.pdf]

Supplemental Material

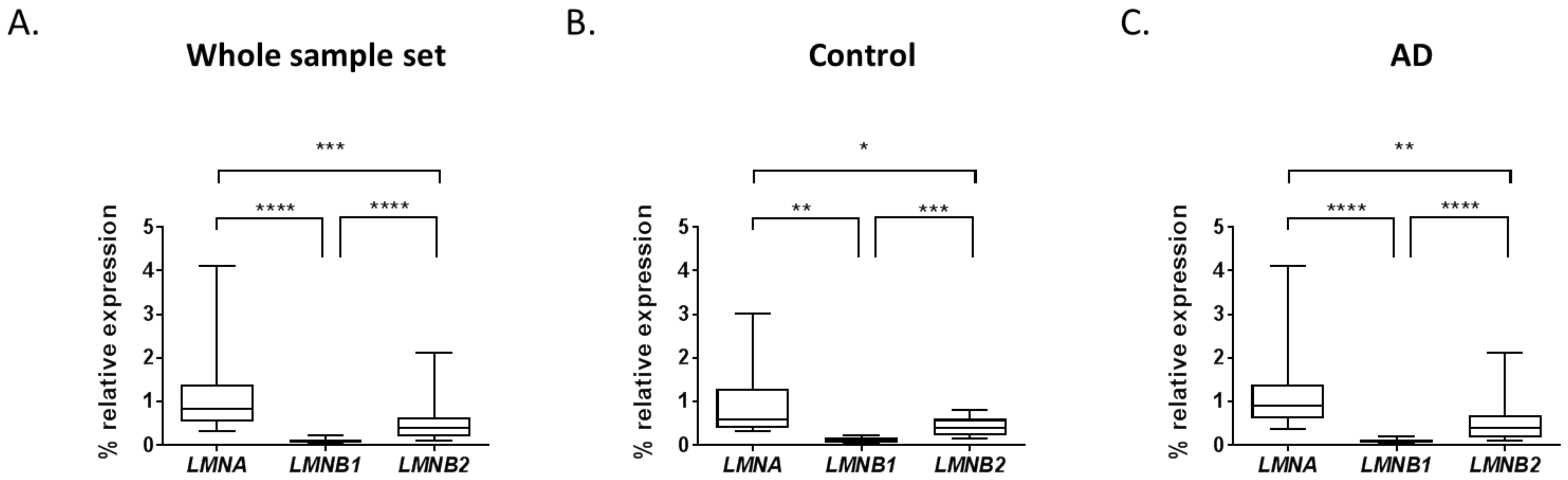

Supplemental Figure S1. Box-plot of percentage of relative expression values of LMNA, LMNB1 and LMNB2 genes.

Whole sample set (AD patients and controls), B) control samples and C) AD samples boxplots show percentage of relative expression values for each lamin genes normalized to GAPDH and ACTB geometric mean. The box for each gene represents the interquartile range (Q1-Q3) of the % relative expression. The line within the box is the median. Whiskers represent the maximum and the minimum values. *p-value < 0.05; **p-value < 0.01; ***p-value < 0.001; *** p-value< 0.0001

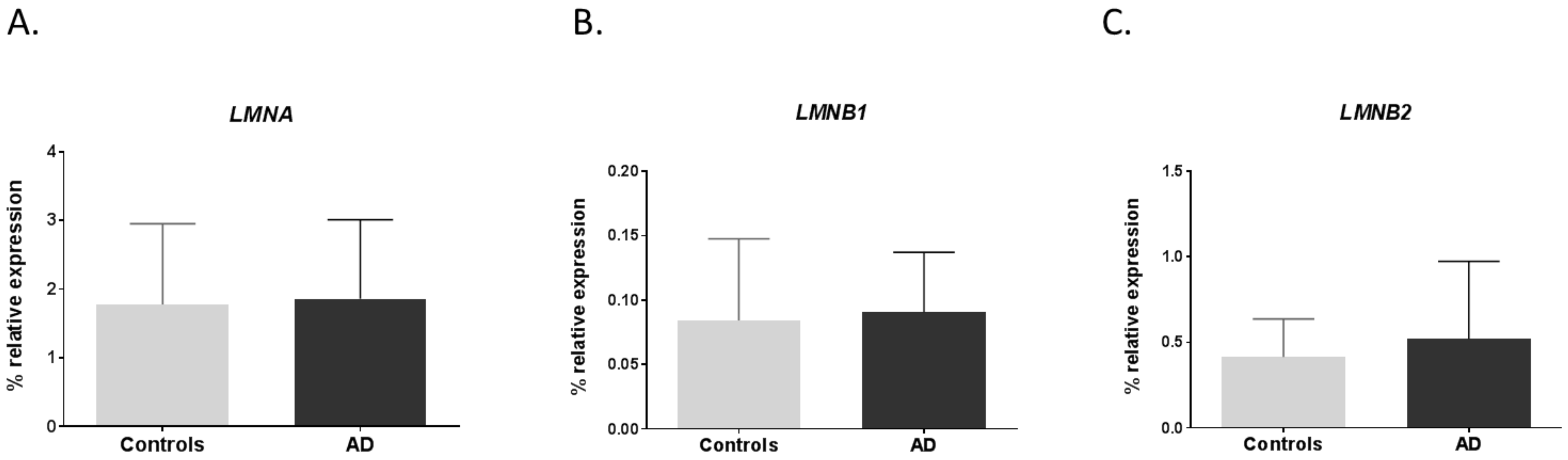

Supplemental Figure S2. mRNA levels of lamin genes in AD and control groups. mRNA expression levels are shown for *LMNA* (A), *LMNB1* (B), and *LMNB2* (C) genes by bar-graphs. Boxes represent percentage of lamin genes expression relative to the geometric mean of GAPDH and ACTB housekeeping genes expression levels. Bars represent the standard error of the mean.

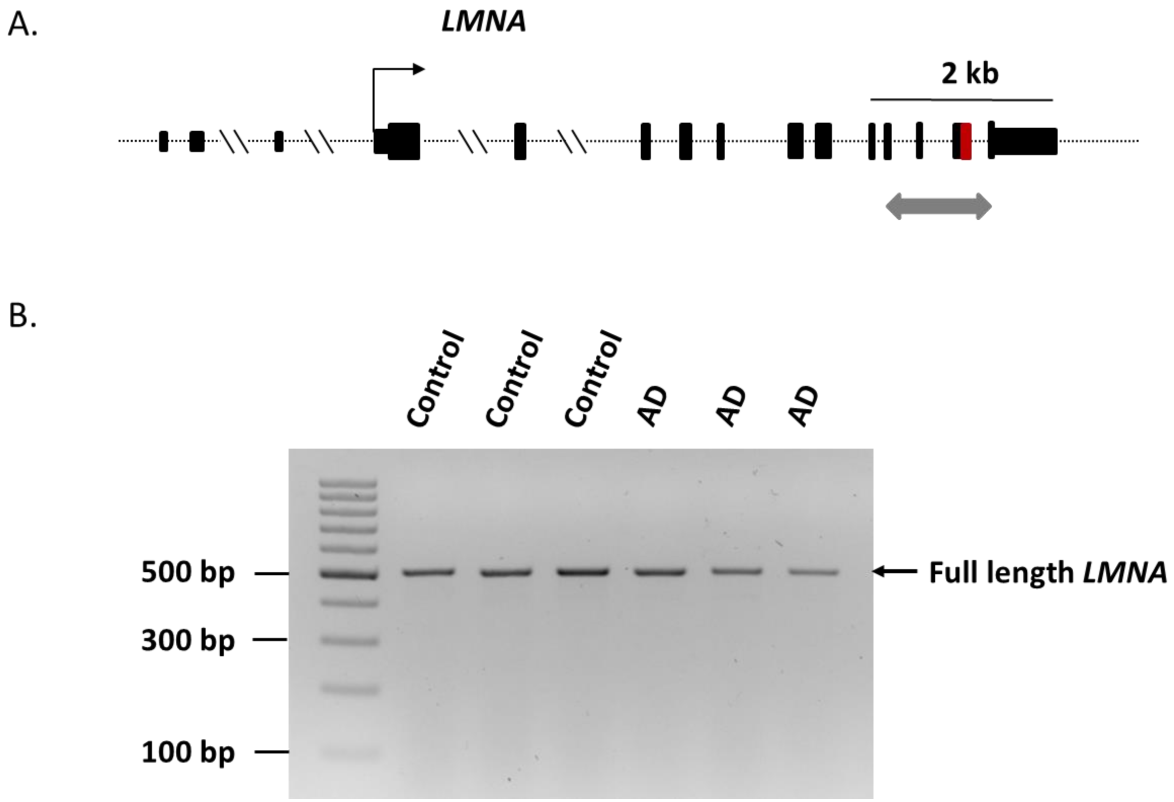

Supplemental Figure S3. Agarose gel electrophoresis of Reverse Transcription-PCR products by using primers spanning *LMNA* exon 9-12.

A. The panel shows *LMNA* transcript map (12 exons). Exons are represented by black boxes and transcription start site by an arrow. The red box denotes the 150 bp deletion characteristic of the progerin transcript. Grey arrow-box represents the 9-to-12 exon amplicon designed to check for the progerin transcript by RT-PCR. **B.** The figure shows a single band corresponding to the full-lenght *LMNA* transcript (510 bp). No amplification for the progerin trancript was observed (360 bp) in the human hippocampus.
